# Psychometric Evaluation of Eating Behaviors and Mental Health Among University Students in China and Pakistan: A Cross-Cultural Study

**DOI:** 10.3390/nu17050795

**Published:** 2025-02-25

**Authors:** Muhammad Waseem Shah, Qinyu Yan, Da Pan, Guiju Sun

**Affiliations:** Key Laboratory of Environmental Medicine Engineering of Ministry of Education, Department of Nutrition and Food Hygiene, School of Public Health, Southeast University, Nanjing 210009, China; shah.mw87@yahoo.com (M.W.S.); yanqinyu1216@outlook.com (Q.Y.); pantianqi92@foxamil.com (D.P.)

**Keywords:** food neophobia, psychometric evaluation, dietary habits, psychological well-being, eating disorders, mental health assessment, orthorexia nervosa, night eating syndrome, depressive symptoms, perceived stress

## Abstract

Background/Objectives: Eating disorders, including food neophobia and restrictive eating behaviors, are pervasive among university students. This study evaluated the psychometric properties of the Nine-Item Avoidant/Restrictive Food Intake Disorder Scale (NIAS), Düsseldorf Orthorexia Scale (DOS), Night Eating Syndrome Questionnaire (NESQ), Zung’s Self-Rating Depression Scale (SDS), and Perceived Stress Scale (PSS) among university students in China and Pakistan to assess reliability and validity and explore the relationships between eating behaviors and mental health. Methods: Initially, responses from 1056 university students from China and Pakistan were obtained, which were reduced to 1002 after screening. Sociodemographic data were collected. Descriptive statistics, reliability analysis, and Confirmatory Factor Analysis (CFA) were performed using SPSS and Analysis of Moment Structures (AMOS). Results: The demographic data showed that 52% of the participants were female and 48% were male, with a mean age of 22.13 ± 3.86 years. Most were single (93.2%) and had a mean BMI of 22.06 ± 4.02 kg/m^2^. The NIAS showed high internal consistency (Cronbach’s Alpha: 0.731), and the CFA revealed strong factor loadings (0.57–0.79). The DOS showed good psychometric properties (factor loadings: 0.53–0.77). The NESQ indicated higher night eating behaviors in Chinese students, who also had higher stress (PSS: Mean Difference = 4.116, 95% CI: 3.36–4.87) and depression (SDS: Mean Difference = 0.229, 95% CI: 0.19–0.27) compared to Pakistani students, who showed more restrictive eating behaviors (NIAS: Mean Difference = −0.422, 95% CI: −0.51 to −0.33). Conclusions: The psychometric evaluation demonstrated strong reliability and validity in assessing eating behaviors and mental health among university students in China and Pakistan. These findings highlight cultural differences, with Chinese students showing higher levels of stress and depression and Pakistani students exhibiting more restrictive eating behaviors. These results suggest the need for culturally tailored interventions to address food-related mental health issues and improve students’ well-being.

## 1. Introduction

Eating behaviors and food preferences play crucial roles in an individual’s overall health and quality of life. These behaviors are influenced by various physiological, psychological, and social factors and significantly impact both physical and mental health outcomes [1]. Among university students, these behaviors are particularly important as they face the dual challenges of academic stress and social change, which can contribute to maladaptive eating patterns and mental health issues [2]. Research has shown that eating disorders, including food neophobia and restrictive eating behaviors, are increasingly prevalent among university populations worldwide [3]. These conditions not only contribute to nutritional deficiencies but also lead to higher rates of stress, depression, and anxiety, which can affect academic performance and overall well-being [4].

Food neophobia, defined as a reluctance to try new or unfamiliar foods, and restrictive eating behaviors as defined by Pliner and Hobden (1992) are considered to be biological mechanisms that affect people’s food choices [5] and are particularly common among university students [6,7,8]. The concept of food neophobia encompasses both the observable actions of rejecting unfamiliar foods and an underlying predisposition to avoid new foods [9]. These actions are typically indicative of underlying psychological challenges, particularly anxiety and depressive disorders, which may contribute to poor dietary habits in adolescents. Studies suggest that food neophobia affects a significant proportion of young adults, with prevalence rates ranging from 10% to 30% [10]. University students, who are exposed to new environments and social pressures, are particularly at risk of developing food-related psychological disorders, including restrictive eating behaviors and orthorexia, a condition characterized by an unhealthy preoccupation with consuming only “healthy” foods [11].

In China and Pakistan, the prevalence of restrictive eating behaviors and food neophobia among university students is a growing concern. Research conducted in China has shown that approximately 15% to 20% of university students suffer from orthorexia, which reflects a strong emphasis on eating only healthy foods, often leading to nutritional imbalances [12]. In addition, mental health issues such as stress and depression are highly prevalent among Chinese university students, with studies indicating that approximately 40% reported symptoms of these conditions during their academic years [13].

Similarly, in Pakistan, food-related psychological disorders are increasingly observed, with studies indicating that a significant proportion of university students exhibit selective eating behaviors or food neophobia [14]. While there is limited research on food neophobia specifically among Pakistani students, studies from South Asia suggest that approximately 25% of students experience restrictive eating patterns [15]. Cultural factors, such as family dynamics and societal expectations, play a significant role in shaping food choices in this population.

Cultural differences, particularly those related to food-related behaviors, play a crucial role in shaping eating patterns and influencing mental health outcomes. According to Hofstede’s cultural dimensions theory, societies vary significantly in the way they structure values, which can directly influence behaviors, including those related to food [16]. In China, the culture is characterized by high power distance and collectivism, where family and social hierarchies strongly impact individual behavior. This societal context may result in a stronger focus on collective harmony, potentially influencing college students’ dietary decisions. This could lead to more homogeneous eating patterns and the internalization of social expectations regarding what constitutes “healthy” eating habits [17].

Conversely, while Pakistan also embraces collective values, their manifestation differs due to unique family dynamics, religious convictions, and economic conditions. In Pakistan, food choices and eating habits are often shaped by the significant influence of family and religious principles [18]. These factors frequently lead to particular dietary preferences and restrictive eating patterns. The cultural factors involved help explain why Pakistani students show higher levels of food neophobia and avoidant eating behaviors. Although both Pakistan and China value collectivism, the particular ways this cultural characteristic is expressed through societal expectations, familial customs, or religious traditions vary considerably between the two countries. These distinctions have a unique impact on the eating habits in each culture [19].

Although food neophobia and restrictive eating patterns are well known, the psychometric characteristics of the Nine-Item Avoidant/Restrictive Food Intake Disorder Scale (NIAS), Düsseldorf Orthorexia Scale (DOS), Night Eating Syndrome Questionnaire (NESQ), Zung’s Self-Rating Depression Scale (SDS), and Perceived Stress Scale (PSS) have been extensively studied in diverse populations [20,21,22,23,24]. These scales were developed through rigorous processes based on both theoretical frameworks and previous research, and their psychometric properties, including reliability and validity, have been established. For instance, the NIAS was developed based on the DSM-5 criteria and the work of Pliner and Hobden (1992) on food neophobia, with its items validated in clinical and non-clinical populations [25,26]. The DOS, designed to assess orthorexia nervosa, follows the framework developed by Donini et al. (2004) and has been used to evaluate orthorexic tendencies in multiple cultural contexts [27,28]. Similarly, the NESQ was constructed to measure night eating behaviors [29], and the SDS and PSS are widely used to assess depression and stress, respectively, with validated factor structures and reliability in diverse populations [30,31,32,33].

However, there remains a gap in the psychometric evaluation of these scales in university students from non-Western contexts, such as China and Pakistan. This study aims to fill this gap by evaluating the validity and reliability of these scales in these cultural contexts, with an emphasis on understanding the relationship between eating behaviors and mental health outcomes such as stress and depression.

The main objectives of this study were as follows:

- To evaluate the validity and reliability of the NIAS, DOS, NESQ, SDS, and PSS scales among university students in China and Pakistan.
- To examine the relationships between food neophobia, restrictive eating behaviors, and psychological health (depression and stress).
- This study aimed to provide insights into the cultural applicability of these scales and their potential for use in cross-cultural comparisons of eating behaviors and mental health issues.

By exploring these relationships, this study will contribute to the development of culturally sensitive interventions aimed at improving eating behaviors and psychological well-being among university students.

## 2. Materials and Methods

### 2.1. Participants

This study initially included 1056 university students from China and Pakistan. The inclusion criteria required participants to be currently enrolled in a university and willing to participate. The exclusion criteria included students who were unwilling to participate or provided incomplete responses to the questionnaires, as incomplete data could introduce bias into the analysis. After screening and data cleaning, the final sample consisted of 1002 participants in total. The sample was composed of 48% male and 52% female students, with a mean age of 22.13 (±3.86) years.

### 2.2. Procedure

The Nine-Item Avoidant/Restrictive Food Intake Disorder Scale (NIAS), Düsseldorf Orthorexia Scale (DOS), Night Eating Syndrome Questionnaire (NESQ), Zung’s Self-Rating Depression Scale (SDS), and Perceived Stress Scale (PSS) were used in this study. These scales and questionnaires are publicly available online and were used according to standard academic practices for the collection of secondary data. This study was approved by the Institutional Review Board (IRB) of Southeast University on 9 July 2024.

Data collection for the Pakistani sample was conducted between July and August 2024 at the University of Agriculture Peshawar. Initially, 500 participants were surveyed for this study. After screening for incomplete responses and missing data, the final valid sample from Pakistan comprised 481 participants. Data collection for the Chinese sample was conducted between October and November 2024. Initially, 556 participants completed the survey. After screening for incomplete responses and missing data, the final valid sample from China comprised 521 participants.

The scales were translated into Chinese for the Chinese participants, and the translation was reviewed by a bilingual, clinical psychologist. The final version of the scales was validated with a small pilot group of students from both countries, and any misunderstandings were corrected. Data were collected through self-administered questionnaires distributed in person to university students. Informed consent was obtained from all participants before they completed the questionnaire. Participants were assured that their responses would remain confidential and that their participation was voluntary and anonymous. The questionnaires included questions on age, sex, height, weight, and the aforementioned scales. No payments or incentives were provided for the participation.

### 2.3. Measures

#### 2.3.1. Nine-Item Avoidant/Restrictive Food Intake Disorder Scale (NIAS)

The NIAS is a self-report scale designed to assess symptoms of avoidant/restrictive food intake disorder (ARFID). The NIAS measures food neophobia, which is characterized by an aversion to trying new or unfamiliar foods. This assessment is conducted through questionnaire items that evaluate avoidance behaviors and fearful reactions towards unfamiliar food items [6]. The scale consists of nine items divided into three subscales: Picky Eating, Appetite, and Fear. Items were rated on a 6-point Likert scale ranging from 0 (Strongly Disagree) to 5 (Strongly Agree). The total score was calculated by summing up the responses, with higher scores indicating more severe avoidant/restrictive eating behaviors.

#### 2.3.2. Düsseldorf Orthorexia Scale (DOS)

The DOS is used to assess orthorexia, a condition characterized by an obsessive focus on eating only healthy foods. Orthorexia is characterized as a strict eating pattern centered on foods perceived as healthy, which adversely impacts both psychological and physical well-being. The scale includes eight items rated on a 5-point Likert scale from 1 (Never) to 5 (Always). Higher scores indicate a stronger tendency toward orthorexic behaviors [34,35].

#### 2.3.3. Night Eating Syndrome Questionnaire (NESQ)

The NESQ is a self-report measure used to assess Night Eating Syndrome (NES), which involves excessive food intake during the night and is associated with stress, depression, and sleep disturbances. The NESQ operationalizes Night Eating Syndrome by using measures that evaluate nocturnal eating practices and the emotional aspects of eating at night [36]. The scale includes several items rated on a 5-point Likert scale from 1 (Never) to 5 (Always).

#### 2.3.4. Zung’s Self-Rating Depression Scale (SDS)

The SDS is a widely used scale to assess symptoms of depression. Depression is defined as a persistent feeling of sadness or a lack of interest in usual activities. The SDS implements this concept through various items that measure how often depressive symptoms occur. The scale consists of 20 items rated on a 4-point Likert scale from 1 (None or Little of the Time) to 4 (Most of the Time). The total score is used to assess the severity of depressive symptoms [37].

#### 2.3.5. Perceived Stress Scale (PSS-14)

The PSS is a self-report measure that assesses the perceived stress levels of individuals. An individual’s thoughts or feelings regarding the level of stress they are experiencing at a certain moment or over a specific time period are known as perceived stress. This is operationalized by the PSS through items that assess feelings of unpredictability, uncontrollability, and overload. The scale consists of 14 items, with responses rated on a 5-point Likert scale from 0 (Never) to 4 (Very Often). Higher scores indicate greater levels of perceived stress [38].

### 2.4. Data Analysis

Data were analyzed using SPSS version 27.0, Excel, and AMOS version 24.0. Descriptive statistics, including the means and standard deviations, were calculated for all variables. An Independent Samples *t*-test was performed to assess the differences between groups (e.g., gender and nationality) in the total scores of the scales. Levene’s test was used to check for homogeneity of variances, and *t*-values, degrees of freedom (df), *p*-values, and Mean Differences were reported to determine the significance of the results. A *t*-test was used to evaluate cultural differences between Chinese and Pakistani students on scales measuring eating behaviors (NIAS, DOS) and psychological health (SDS, PSS).

Confirmatory Factor Analysis (CFA) was used to evaluate the construct validity of the scales, with fit indices such as CFI, TLI, and RMSEA being used to assess model fit. The reliability of the scales was assessed using Cronbach’s Alpha coefficients, with values above 0.70 indicating acceptable internal consistency. Item-total correlations were calculated to assess the quality and consistency of individual items within each scale. ANOVA was performed to examine differences in eating behaviors and psychological health based on demographic variables. Post hoc tests (Tukey’s HSD test) were conducted to determine the source of significant differences. Mann–Whitney U tests for BMI categories were also performed (Supplementary Materials).

## 3. Results

### 3.1. Sociodemographic Characteristics

The sample comprised 1002 university students (Table 1), with a nearly equal gender distribution (48% male and 52% female). Participants were from China (48%) and Pakistan (52%), reflecting the focus of the study on these two populations. The majority of participants were single (93.2%), followed by married (6.7%), and a small proportion were divorced (1%) participants. In terms of education, most participants were pursuing a bachelor’s degree (74.7%), with fewer pursuing master’s (17.4%) and PhD (8%) degrees.

The mean age of the participants was 22.13 ± 3.86 years. The mean body weight was 61.11 ± 12.74 kg and the mean height was 166.17 ± 8.79 cm. Regarding BMI (Body Mass Index), 65.8% of participants had a normal weight (BMI: 18.5–24.9 kg/m^2^), while 15.5% were classified as underweight, and 15.6% were classified as overweight or obese. The mean BMI was 22.06 ± 4.02 kg/m^2^.

### 3.2. Descriptive Statistics and Reliability of the NIAS Scale

Table 2 presents the descriptive statistics, reliability analysis, and CFA results for the NIAS. The mean scores for the items range from 0.71 to 1.5, indicating that the behaviors measured by the scale are generally infrequent in the sample. The standard deviation values varied moderately across the items, with the highest standard deviation of 1.43 for NIAS 1 (Picky Eating), suggesting a wider range of responses for this item.

The item-total correlations range from 0.53 to 0.70, showing that each item meaningfully contributes to its respective subscale. Items like NIAS 7 have a higher correlation of 0.79, indicating a stronger relationship with the overall construct. On the other hand, NIAS 2, with a correlation of 0.53, still shows a relevant contribution to the scale despite having a lower value.

The factor loadings for each item ranged from 0.57 to 0.79, indicating that all items made a meaningful contribution to the underlying factors they were intended to measure. The NIAS 7 (0.79) and NIAS 9 (0.76) items showed a strong relationship with their respective factors, while NIAS 2 (0.57) showed a weak relationship but still remained within an acceptable range.

The Cronbach’s Alpha values for each subscale ranged from 0.61 to 0.76. The Picky Eating subscale had a Cronbach’s Alpha of 0.61, which indicates acceptable internal consistency. The Appetite subscale showed good internal consistency, with a Cronbach’s Alpha of 0.71, and the Fear subscale had the highest Cronbach’s Alpha of 0.76, indicating strong internal consistency. The overall reliability of the full scale, combining all three subscales (Picky Eating, Appetite, and Fear), was 0.731, which suggests acceptable internal consistency for the NIAS scale as a whole.

### 3.3. Confirmatory Factor Analysis of NIAS

Table 3 presents the CFA goodness-of-fit indices for the NIAS. The model fit indices indicated that the CFA model fits the data well. The CFI value was 0.966, which exceeds the typical threshold of 0.90, which suggested a very good fit. The Tucker–Lewis Index (TLI) of 0.947 was above the acceptable threshold of 0.90, indicating a good model fit. The Root Mean Square Error of Approximation (RMSEA) value of 0.061, which was below the recommended maximum of 0.08, suggested a good fit with the data. The Chi-Square Minimum Degrees of Freedom (CMIN/DF) value of 4.678, although slightly above the ideal threshold of 3, still falls within the acceptable range according to the Structural Equation Modeling (SEM) guidelines. These indices reflect the standard thresholds used in Confirmatory Factor Analysis (CFA) to evaluate model fit, which are widely accepted in the literature (e.g., Comparative Fit Index (CFI) and TLI ≥ 0.90, RMSEA ≤ 0.08, and CMIN/DF ≤ 3).

### 3.4. AMOS Model Fit Diagram for the NIAS

**Figure 1:**
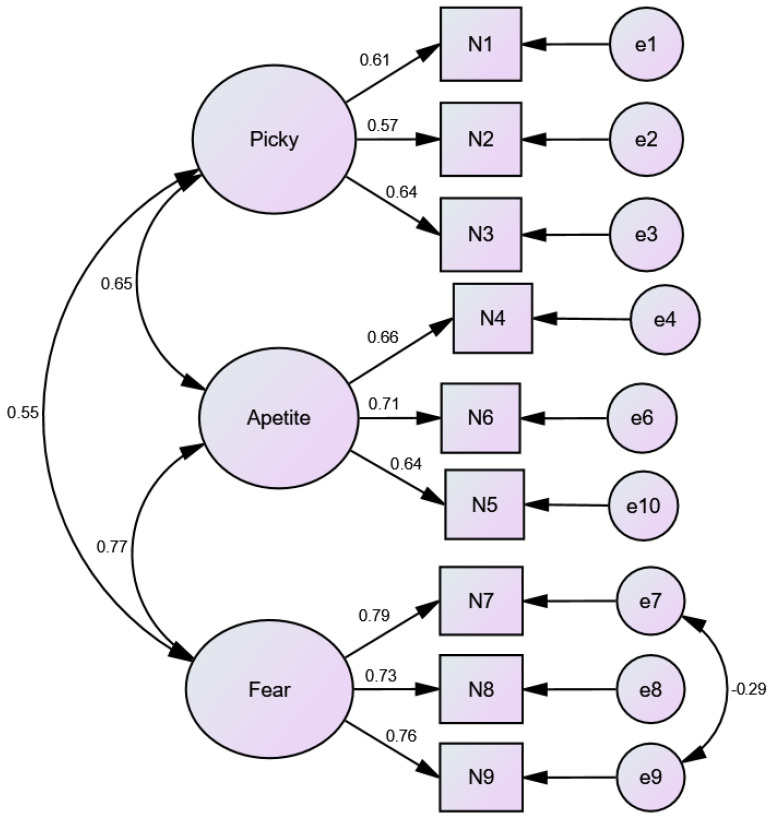
AMOS model fit diagram for the Nine-Item Avoidant/Restrictive Food Intake Disorder Scale (NIAS).

Figure 1 illustrates the factor structure of the NIAS, which comprises three distinct factors: Picky Eating, Appetite, and Fear. Each factor is represented by several items, with the factor loadings shown as the strength of the relationship between each item and its respective factor. For instance, the Fear factor has relatively high factor loadings on items such as N7, N8, and N9, suggesting that these items are strongly related to the underlying Fear dimension of selective eating behaviors. Similarly, the Appetite factor includes items such as N4, N5, and N6, reflecting the relationship between appetite-related symptoms and the overall construct.

### 3.5. CFA and Model Fit Indices for Düsseldorf Orthorexia Scale (DOS)

The Confirmatory Factor Analysis (CFA) for the eight-item Düsseldorf Orthorexia Scale (DOS), as shown in Table 4, demonstrated that most items contributed effectively to the overall construct of orthorexia-related behaviors. The factor loadings for most items ranged from 0.487 to 0.659, with items such as D3 and D4 showing strong contributions to the overall construct. According to standard practices, a factor loading of 0.50 or higher is considered acceptable, with values closer to 0.60 or above indicating a stronger contribution to the factor structure. Items D5 and D6 showed slightly weaker loadings but remained within an acceptable range. Item D9 showed a factor loading of 0.534, which is also within the acceptable range, while D10 had a loading of 0.587, reflecting a moderate contribution to the factor structure.

The model fit indices indicate a good fit, with a CFI of 0.975, TLI of 0.959, and RMSEA of 0.053, all meeting the recommended thresholds. The SRMR value of 0.03 and PCLOSE value of 0.354 further support the model’s good fit. The Cronbach’s Alpha value of 0.807 indicates strong internal consistency, and the Composite Reliability (CR) value of 0.796 supports the reliability of the scale. However, the Average Variance Extracted (AVE) was 0.33, which is below the recommended threshold of 0.50, indicating room for improvement in the scale’s convergent validity.

### 3.6. AMOS Model Fit Diagram for the 8-Item DOS Scale

**Figure 2:**
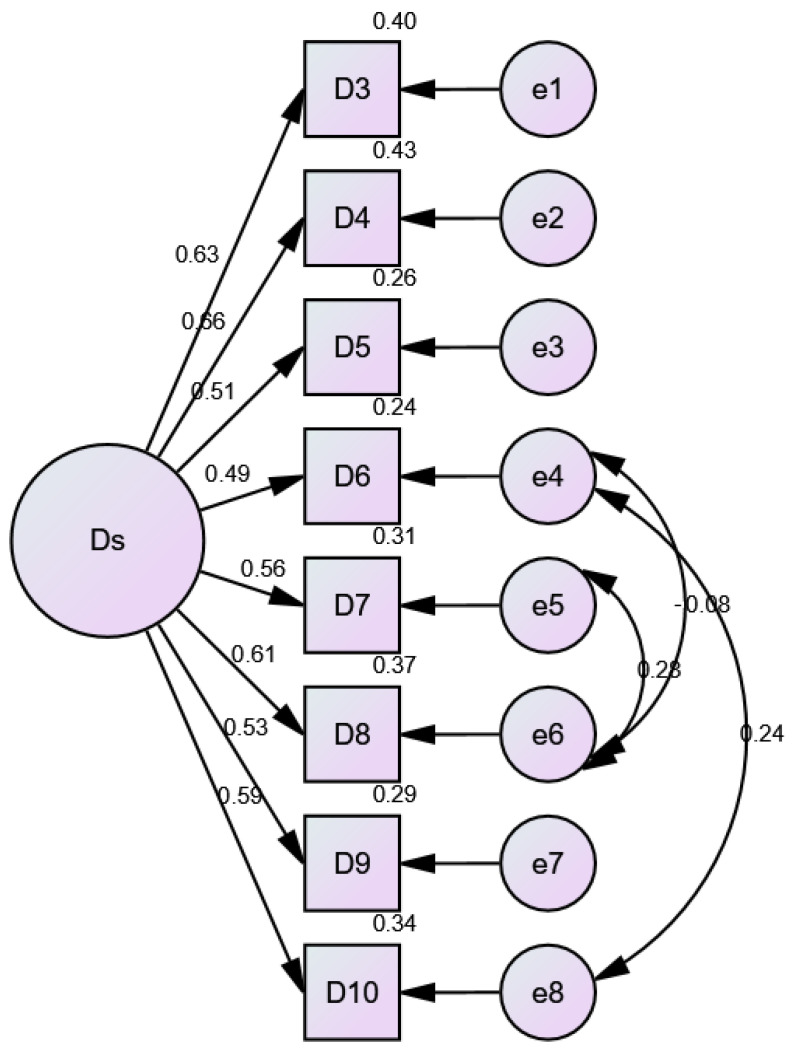
AMOS model fit diagram for DOS 8 items.

Figure 2 displays the factor structure of the DOS, with the single latent factor DS representing orthorexic tendencies. Items like D3 and D4 show relatively strong factor loadings, suggesting that they are more closely related to the underlying construct of orthorexia. Other items, such as D5 and D6, have lower loadings, indicating weaker contributions to the DS factor. This visual representation highlights how the individual items contribute to measuring orthorexic behaviors and attitudes.

### 3.7. Factor Analysis of Night Eating Syndrome Questionnaire (NESQ)

The Factor Analysis of the NESQ revealed two distinct dimensions. Factor 1 represented emotional or behavioral eating habits, with items such as NESQ11 (Factor Loading: 0.813; Communality: 0.661) and NESQ10 (Factor Loading: 0.771; Communality: 0.598) showing strong contributions. This factor explained 23.98% of the variance. Factor 2 captured dietary adherence or restraint behaviors, with items like NESQ9 (Factor Loading: 0.605; Communality: 0.426), NESQ8 (Factor Loading: 0.579; Communality: 0.357), and NESQ6 (Factor Loading: 0.475; Communality: 0.232) contributing substantially. This factor explained 15.22% of the variance. Together, the two factors accounted for 39.2% of the total variance.

The overall reliability of the NESQ scale improved after removing weaker items, resulting in a Cronbach’s Alpha of 0.667 for the remaining seven items. NESQ11 and NESQ10 demonstrated strong corrected item-total correlations (0.421 and 0.470, respectively) and high reliability scores, suggesting their importance in assessing emotional or behavioral eating habits.

However, NESQ5 displayed low communalities (0.151) and weak factor alignment, raising concerns about its contribution to the overall scale. NESQ12, with a factor loading of 0.511 and a commonality of 0.32, showed a moderate contribution to Factor 1, but its lower corrected item-total correlation (0.389) suggested it was not as strong as other items in evaluating emotional or behavioral eating habits (Table 5).

Exploratory Factor Analysis (EFA) was conducted for the NESQ, and the results revealed two factors: emotional/behavioral eating habits and dietary adherence/restraint behaviors. However, the CFA for the NESQ did not meet the required thresholds for model fit (CFI, RMSEA, TLI), and as a result, the CFA results were not included in the manuscript. This issue is explained earlier in the Results section for transparency.

### 3.8. Factor Structure and Reliability of the Zung Self-Rating Depression Scale (SDS)

Table 6 presents the reliability and factor analysis results for SDS. The Core Depressive Symptoms subscale included items such as “I feel hopeful about the future” (standardized factor loading (SFL): 0.815), “I feel useful and needed” (SFL: 0.808), and “My life is pretty full” (SFL: 0.715). These items had high factor loadings, indicating they strongly contributed to the Core Depressive Symptoms factor. The Cronbach’s Alpha if any of these items were deleted ranged from 0.66 to 0.663, suggesting good internal consistency for the subscale.

The Cognitive Symptoms subscale included items like “I find it easy to do things” (SFL: 0.678) and “My mind is as clear as it used to be” (SFL: 0.611). These items had moderate factor loadings, with the Cronbach’s Alpha if any of these items were deleted ranging from 0.654 to 0.666, indicating acceptable internal consistency.

The Anxiety Symptoms subscale included “I have trouble sleeping at night” (SFL: 0.531), which had a relatively lower factor loading compared to other items. The Cronbach’s Alpha if this item was deleted was 0.673, showing moderate reliability.

The Somatic Symptoms subscale included “My heart beats faster than usual” (SFL: 0.385) and “I get tired for no reason” (SFL: 0.71). The factor loadings for these items varied, with the second item having a much stronger relationship to the Somatic Symptoms factor. The Cronbach’s Alpha if any of these items were deleted was 0.681, reflecting moderate internal consistency. The overall Cronbach’s Alpha for the full 16-item scale was 0.721, which indicated good internal consistency across the scale.

The EFA for Zung’s Self-Rating Depression Scale identified several meaningful subscales associated with depressive symptoms, cognitive effects, anxiety, and somatic experiences. Unfortunately, the CFA results did not meet the acceptable model fit indices (CFI, RMSEA, and TLI); thus, these results were not included in the manuscript.

### 3.9. Reliability and Stress Level Categorization of Perceived Stress Sale (PSS 9 Items)

Table 7 presents the stress level categorization and reliability of the 9-item Perceived Stress Scale (PSS). Stress levels were divided into three categories: low stress (0–13), moderate stress (14–26), and high stress (27–40). According to the results, the majority of participants (63.2%) fell into the moderate stress category, reporting scores between 14 and 26. Meanwhile, 29.1% of the participants were categorized as experiencing high stress, with scores between 27 and 40. Only 7.7% of the participants had low stress, with scores between 0 and 13. The reliability of the PSS-9 was also assessed, with a Cronbach’s Alpha of 0.743, indicating good internal consistency. This suggests that the scale is a reliable measure for assessing perceived stress across various stress levels.

### 3.10. Association Between PSS Stress Categories and Demographic Variables

Table 8 presents the crosstab analysis results for the association between PSS stress categories and gender, nationality, and education level. Among the male participants, 61.7% reported moderate stress, 30.3% had high stress, and 8% had low stress. Among the female participants, 64.2% reported moderate stress, 28.3% reported high stress, and 7.5% reported low stress. The *p*-value for gender was not statistically significant (N.S.), indicating no significant difference between males and females in terms of stress levels.

Regarding nationality, 71.8% of the Chinese participants reported moderate stress, 16.5% had high stress, and 11.7% had low stress. Among the Pakistani participants, 53.8% had moderate stress, 42.8% experienced high stress, and 3.3% had low stress. The *p*-value for nationality was less than 0.001, indicating a significant association between nationality and stress levels.

In terms of education level, 65.2% of bachelor’s students reported moderate stress, 25.9% had high stress, and 8.8% had low stress. Among master’s students, 62.1% reported moderate stress, 33.3% had high stress, and 4.6% had low stress. Among the doctoral students, 46.3% reported high stress, 46.3% had moderate stress, and 3.8% had low stress. The *p*-value for education level was less than 0.001, indicating a significant association between education and stress levels.

### 3.11. Factor Analysis and Reliability of the Perceived Stress Scale Factors (PSS-9 Items)

Table 9 presents the factor analysis and reliability results for PSS-9. The Perceived Helplessness subscale, which included seven items (P4, P5, P6, P7, P9, P10, and P13), demonstrated good internal consistency, with a Cronbach’s Alpha value of 0.891. This subscale accounted for 45.09% of the total variance. The Self-Efficacy subscale, consisting of two items (P1 and P3), showed a moderate inter-item correlation of 0.609. However, Cronbach’s Alpha was not applicable (N/A) for this subscale, likely because of the small number of items (only two), which makes it challenging to calculate a reliable alpha coefficient. The Self-Efficacy subscale explained 12.75% of the total variance.

Exploratory Factor Analysis (EFA) and Confirmatory Factor Analysis (CFA) were conducted on the PSS-9 scale to evaluate its underlying structure. The EFA revealed two dimensions: Perceived Helplessness and Self-Efficacy, accounting for 45.09% and 12.75% of the total variance, respectively. The reliability for each subscale was confirmed with high Cronbach’s Alpha for Perceived Helplessness (0.891), while the self-efficacy subscale did not yield an applicable Cronbach’s Alpha due to its limited number of items. CFA was also performed to verify the model fit, with the indices showing an acceptable fit for the two-factor structure of the PSS scale.

### 3.12. Cultural Differences in Eating Patterns and Psychological Health

Table 10 presents significant differences in eating behaviors and psychological health between Chinese and Pakistani students. Pakistani students scored higher on the NIAS total score, indicating a greater tendency toward avoidant and restrictive eating behaviors. In contrast, Chinese students scored higher on DOS, SDS, and PSS.

The Independent Samples *t*-test revealed significant differences in key psychological and eating behavior scales between the two student groups. Pakistani students exhibited higher NIAS total scores (Mean Difference = −0.422, 95% CI: −0.51472 to −0.32946), reflecting more pronounced avoidant/restrictive eating behaviors. On the other hand, Chinese students demonstrated higher DOS total scores (Mean Difference = 0.335, 95% CI: 0.27488 to 0.39556), suggesting more significant orthorexic tendencies. Additionally, Chinese students showed higher SDS total scores (Mean Difference = 0.229, 95% CI: 0.18556 to 0.27326), indicating greater depressive symptoms, and higher PSS total scores (Mean Difference = 4.116, 95% CI: 3.35896 to 4.87218), showing significantly higher stress levels.

Levene’s test results indicated that the assumption of homogeneity of variance was violated for the NIAS (F = 49.506, *p* < 0.001), DOS (F = 132.836, *p* < 0.001), SDS (F = 12.391, *p* < 0.001), and PSS (F = 7.282, *p* = 0.007), suggesting unequal variances between the groups. Despite this violation, the Independent Samples *t*-test was still conducted. The results showed significant differences between the groups, providing important insights into the psychological and eating behavior patterns of Chinese and Pakistani students.

## 4. Discussion

This present study aimed to evaluate the psychometric properties of several scales measuring eating behaviors and mental health among university students in China and Pakistan, while also exploring cross-cultural differences in these behaviors. The findings provide valuable insights into the prevalence of avoidant/restrictive eating behaviors, orthorexic tendencies, night eating syndrome, depressive symptoms, and perceived stress among university students in these two countries. The results provide valuable insights into the reliability and validity of the scales used, offering a robust foundation for future research in this area and also highlighting the implications of these results for understanding eating behaviors and mental health in cross-cultural contexts.

This study contributes to the theoretical understanding of eating behaviors and mental health by extending the application of established scales to non-Western university populations, particularly in China and Pakistan. The psychometric evaluation of the NIAS, DOS, NESQ, SDS, and PSS enhances the cross-cultural validity of these tools. By assessing the applicability of these scales in diverse cultural contexts, this study underscores the role of cultural norms in shaping eating behaviors and psychological well-being [39].

The results revealed that cultural differences in dietary practices, academic pressures, and social expectations significantly influenced eating behaviors and mental health outcomes. To further understand these cultural differences, we reference Hofstede’s cultural dimensions theory [40,41], which compares cultures based on several factors, such as individualism vs. collectivism, power distance, and uncertainty avoidance. According to Hofstede’s model, China is considered a high-power-distance society with strong collectivist values, where respect for authority and group harmony are prioritized [42]. This cultural framework may explain why the Chinese students in our study showed stronger tendencies toward orthorexia, as their eating behaviors may be influenced by social expectations and the desire to conform to health and fitness norms [43]. On the other hand, Pakistan also holds collectivist values but its expression is shaped by family dynamics, religious beliefs, and socioeconomic factors [44,45]. These influences may explain the higher levels of avoidant/restrictive eating behaviors observed in Pakistani students. While both cultures share collectivism, family structures, and socioeconomic factors, these factors likely shape eating behaviors differently, influencing how dietary patterns are followed. These cultural differences indicate that stress, depression, and eating behaviors are strongly influenced by social and familial factors.

Pakistani students showed higher levels of avoidant/restrictive eating behaviors, which may be influenced by cultural dietary practices and socioeconomic factors [46], while Chinese students exhibited stronger tendencies toward orthorexia, reflecting a growing focus on healthy eating and fitness [47]. These findings contribute to a deeper understanding of how stress and depression are intertwined with eating behaviors, offering a theoretical basis for culturally sensitive interventions aimed at improving mental health and eating habits in university populations [48,49]. This study fills an important gap in the literature, providing new insights into the cross-cultural applicability of psychological and eating disorder scales.

The sociodemographic analysis revealed that the majority of participants were single, pursuing a Bachelor’s degree, and had a normal BMI. This is consistent with previous studies that have shown university students often fall within the normal BMI range but are at risk for disordered eating behaviors due to academic stress and lifestyle changes [3,50]. The nearly equal gender distribution in our sample allowed for a balanced analysis of gender differences in eating behaviors and mental health, although no significant gender differences were found in stress levels. This contrasts with some previous studies that have reported higher stress levels among female students [51]. The lack of gender differences in stress levels in our study may be attributed to the unique cultural contexts of China and Pakistan, where both male and female students face significant academic and societal pressures.

The NIAS demonstrated acceptable internal consistency and strong factor loading, with Cronbach’s Alpha values ranging from 0.61 to 0.76 across its subscales. The subscales (Picky Eating, Appetite, and Fear) were particularly well represented, supporting the reliability of the scale for both Chinese and Pakistani students. The factor structure of the NIAS was confirmed through Confirmatory Factor Analysis (CFA), with good model fit indices (CFI = 0.966, RMSEA = 0.061). These findings align with previous research that has validated the NIAS as a reliable tool for assessing avoidant/restrictive eating behaviors [52].

Regarding the presentation of the models, we chose to present separate models for each scale (NIAS, DOS, NESQ, SDS, and PSS) rather than combining all items into a single model. This decision was made to preserve the theoretical integrity of each scale, as each is designed to measure distinct constructs. The NIAS assesses avoidant/restrictive eating behaviors, the DOS measures orthorexic tendencies, and the NESQ, SDS, and PSS focus on psychological factors such as stress and depression. Combining all items into one model could compromise the clarity of these constructs and reduce the specificity of the analysis, as suggested by previous psychometric research [33,53]. Factor models should ideally reflect the theoretical structure of the scale being measured, and grouping items from distinct constructs may lead to less interpretable results [54]. By maintaining separate models, we ensure that each scale is evaluated independently, reflecting the unique aspects of eating behaviors and mental health it is intended to measure, which has been recommended in earlier studies on scale validation [55,56]. This approach also allows for more accurate factor analysis and psychometric evaluation for each tool.

The higher scores on the NIAS among Pakistani students suggest a greater prevalence of avoidant/restrictive eating behaviors in this population, which may be linked to cultural dietary practices or socioeconomic factors, and these findings align with existing research suggesting that cultural norms and family dynamics in South Asia significantly influence eating patterns [57]. In Pakistan, the traditional dietary habits and food availability may contribute to more restrictive eating patterns, whereas in China, the emphasis on dietary diversity and health may reduce the prevalence of such behaviors.

The DOS also showed good reliability, with a Cronbach’s Alpha of 0.807. However, the Average Variance Extracted (AVE) was below the recommended threshold of 0.50, indicating room for improvement in the scale’s convergent validity. This is consistent with previous critiques of the DOS, which have noted that while it is a useful tool for measuring orthorexic tendencies, it may require further refinement to improve its psychometric properties [58]. The higher DOS scores among Chinese students suggest a greater tendency toward orthorexic behaviors. This trend may be attributed to cultural perspectives on nutrition and wellness. The findings underscore the significant role that cultural context significantly influences eating behaviors. In China, the growing emphasis on healthy eating and fitness, particularly among young adults, may contribute to orthorexic tendencies. This finding aligns with studies that have highlighted the rise of orthorexia in urbanized, health-conscious populations [59]. Furthermore, societies that strongly prioritize nutritious diets tend to see a higher incidence of orthorexia, as individuals become increasingly focused on consuming exclusively what they believe to be healthy foods [60,61]. This suggests that cultural attitudes towards food play a key role in shaping eating patterns.

The NESQ revealed two distinct dimensions such as emotional/behavioral eating habits and dietary adherence/restraint behaviors. The emotional eating factor, which explained 23.98% of the variance, was strongly associated with items such as eating in response to stress or emotional distress. This is consistent with previous research that has linked night eating syndrome to emotional dysregulation and stress [62]. The dietary adherence/restraint factor, which explained 15.22% of the variance, was associated with items related to dietary control and restraint. This finding aligns with studies that have identified dietary restraint as a key component of night eating syndrome [63]. The higher stress and depression levels observed in Chinese students could explain their increased nocturnal eating tendencies. Emotional distress can lead to more frequent nighttime food consumption as a coping mechanism for negative emotions. The overall reliability of the NESQ scale was acceptable (Cronbach’s Alpha = 0.667), suggesting that it is a useful tool for assessing night eating behaviors in university students. However, the weak contribution of some items (e.g., NESQ5) indicates that further refinement of the scale may be necessary.

The SDS demonstrated good internal consistency (Cronbach’s Alpha = 0.721), with strong factor loadings for items related to Core Depressive Symptoms. The higher SDS scores among Chinese students indicated a greater prevalence of depressive symptoms in this group, which is consistent with previous research highlighting the high levels of mental health issues among Chinese university students [64,65]. The stress associated with academic performance and societal expectations may contribute to these elevated depressive symptoms. In contrast, Pakistani students reported lower levels of depressive symptoms, which may be attributed to stronger social support networks or cultural differences in the expression of mental health issues [66,67].

The Perceived Stress Scale showed good reliability (Cronbach’s Alpha = 0.743), with the majority of participants reporting moderate-to-high levels of stress. This is consistent with previous studies that found high levels of stress among university students, particularly in Asian countries [68]. Several studies also reported that students in high-pressure academic environments experienced higher levels of stress and anxiety [69,70,71].

The significant differences in stress levels between Chinese and Pakistani students may be attributed to cultural and educational differences, with Chinese students reporting higher stress levels, possibly due to the intense academic competition in China [72]. In contrast, Pakistani students may experience different stressors, such as financial pressures or concerns about future employment, which were not specifically measured in this study.

This current study revealed significant cross-cultural differences in eating behaviors and mental health. Pakistani students scored higher on the NIAS, indicating a greater tendency toward avoidant/restrictive eating behaviors, while Chinese students scored higher on the DOS, SDS, and PSS, reflecting higher levels of orthorexic tendencies, depressive symptoms, and perceived stress. These findings suggest that cultural factors play a significant role in shaping eating behaviors and mental health outcomes. For example, the emphasis on academic achievement in Chinese culture may contribute to higher stress and depressive symptoms, while dietary practices in Pakistan may influence the prevalence of avoidant/restrictive eating behaviors. These findings are consistent with previous research that has highlighted the impact of cultural context on mental health and eating behaviors [73,74].

## 5. Limitations and Strength of the Study

Although this study provides valuable insights into the psychometric properties of NIAS, DOS, NESQ, SDS, and PSS among university students in China and Pakistan, it has several limitations. The cross-sectional design limits the ability to establish causal relationships between eating behaviors and psychological health. The sample, which was restricted to university students, may not fully represent the broader population in these countries, especially in terms of socioeconomic status, geographical diversity, and relationship status. The high percentage of single participants in our sample may limit the generalizability of the findings, as relationship status can potentially influence eating behaviors and mental health outcomes. Additionally, self-reported questionnaires may have introduced social desirability bias or inaccurate reporting, particularly on sensitive topics, such as eating behaviors and mental health. While the scales demonstrated good reliability and validity, their applicability across different cultural contexts requires further validation. This study also excluded participants with incomplete responses, potentially leading to a selection bias. Finally, the findings may not be generalizable to non-university populations or individuals outside the age group of university students.

Despite these limitations, the strengths of this study include robust psychometric analysis, use of validated scales, and cross-cultural comparison. These aspects provide valuable insights into eating behaviors and mental health of university students in China and Pakistan.

## 6. Conclusions and Future Research

The psychometric properties of the NIAS, DOS, NESQ, SDS, and PSS were assessed in university students in China and Pakistan. The results demonstrated that these scales are reliable and valid tools for evaluating eating behaviors and mental health in both populations. Significant cultural differences were observed, with Chinese students exhibiting higher levels of orthorexic tendencies, stress, and depression, whereas Pakistani students showed more pronounced restrictive eating behaviors. These findings underscore the need to consider cultural context when addressing food-related mental health issues among university students. The significance of mental health in influencing dietary patterns is also highlighted, indicating that culturally appropriate strategies are essential for encouraging healthier food choices and enhancing psychological well-being in university environments.

The findings provide crucial insights into the eating behaviors and psychological health of students in both countries. Future research should explore the overlap between eating disorders and other mental health conditions, such as anxiety and body image disorders, to gain a deeper understanding of how these factors interact and influence each other. Additionally, future studies should consider the role of additional cultural and socioeconomic factors that may influence eating behaviors and mental health, such as family dynamics, religious beliefs, and access to mental health services. Longitudinal studies could offer a deeper understanding of how these behaviors evolve over time, especially in relation to academic performance and personal well-being. Expanding the sample to include a broader range of countries and non-university populations would strengthen the generalizability of our findings.

Furthermore, future studies should continue to explore these differences and develop culturally sensitive interventions to address the unique needs of students in different cultural settings. Specific strategies for developing these interventions include tailoring mental health programs to address cultural and social factors influencing eating behaviors, incorporating cultural beliefs and values into eating disorder interventions, providing training for mental health professionals and university staff, and encouraging collaboration between cultural experts, healthcare professionals, and students to create effective and culturally appropriate programs.

## Figures and Tables

**Table 1:** Sociodemographic characteristics of university students from China and Pakistan.

Variable	Category	Frequency (%)
Gender	Male	426 (48)
	Female	576 (52)
Marital Status	Married	67 (6.7)
	Single	934 (93.2)
	Divorced	1 (1)
Nationality	Chinese	481 (48)
	Pakistani	521 (52)
Education	Bachelor’s	748 (74.7)
	Master’s	174 (17.4)
	PhD	80 (8)
Age	Mean ± SD	22.13 ± 3.86
Weight (kg)		61.11 ± 12.74
Height (cm)		166.17 ± 8.79
BMI (kg/m^2^)		22.06 ± 4.02

**Table 2:** Descriptive statistics, reliability analysis, and confirmatory factor analysis for NIAS.

Dimension	Items	Mean	SD ^1^	Item-Total Correlation	SFL ^2^	IIDCα ^3^	Cα ^4^
Picky Eating	NIAS 1	1.5	1.43	0.58	0.61	0.809	0.61
	NIAS 2	0.88	1.07	0.53	0.57	0.806	
	NIAS 3	1.32	1.4	0.62	0.64	0.802	
Appetite	NIAS 4	0.98	1.16	0.67	0.66	0.789	0.71
	NIAS 5	1.13	1.27	0.65	0.64	0.792	
	NIAS 6	1.02	1.2	0.67	0.71	0.788	
Fear	NIAS 7	0.71	1.11	0.7	0.79	0.785	0.76
	NIAS 8	0.85	1.13	0.67	0.73	0.788	
	NIAS 9	0.77	1.07	0.61	0.76	0.796	
Overall Reliability	Picky Eating, Appetite, Fear						0.731

^1^ Standard deviation, ^2^ Standardized factor loading, ^3^ Cronbach’s Alpha (if Item Deleted), ^4^ Cronbach’s Alpha.

**Table 3:** CFA model fit indices for NIAS.

Fit Index	Value	Threshold	Interpretation
CFI	0.966	≥0.90	Excellent fit
CMIN/DF	4.678	<5.0	Acceptable fit
TLI	0.947	≥0.90	Very good fit
RMSEA	0.061	≤0.08	Good fit
SRMR	0.0324	≤0.08	Acceptable fit
PCLOSE	0.06	≥0.05	Close-fitting model

**Table 4:** Factor loadings, error variance, and goodness of fit indices (DOS).

Variable	Items	SFL ^1^	Er. Var ^2^	Fit Index	Value	Threshold
DOS	D3	0.629	0.604	CFI	0.975	≥0.90
	D4	0.659	0.566	CMIN/DF	3.768	≤3
	D5	0.511	0.739	TLI	0.959	≥0.90
	D6	0.487	0.763	RMSEA	0.053	≤0.08
	D7	0.56	0.686	SRMR	0.03	≤0.08
	D8	0.609	0.622	PCLOSE	0.354	≥0.05
Reliability and Validity	Composite Reliability (CR)				0.796	≥0.70
	Average Variance Extracted (AVE)				0.33	≥0.50

^1^ Standardized factor loadings. ^2^ Error variance.

**Table 5:** Reliability and Factor Analysis results for NESQ items.

Item	Factor	SFL ^1^	Communalities	CITC ^2^	IIDCα ^3^
NESQ11	Factor 1	0.813	0.661	0.421	0.617
NESQ10	Factor 1	0.771	0.598	0.47	0.602
NESQ12	Factor 1	0.511	0.32	0.389	0.605
NESQ9	Factor 2	0.605	0.426	0.447	0.613
NESQ8	Factor 2	0.579	0.357	0.411	0.636
NESQ6	Factor 2	0.475	0.232	0.162	0.695
NESQ5	Factor 1	0.151	0.151	0.346	0.642

^1^ Standardized factor loadings, ^2^ Corrected total-item correlation, ^3^ Cronbach’s Alpha (if Item Deleted).

**Table 6:** Reliability and Factor Analysis results for the Zung Self-Rating Depression Scale (SDS).

Subscales	Items	SFL ^1^	Communalities	CITC ^2^	IIDCα ^3^
Core Depressive Symptoms	I feel hopeful about the future	0.815	0.691	0.389	0.663
	I feel useful and needed	0.808	0.665	0.421	0.66
	My life is pretty full	0.715	0.573	0.42	0.661
Cognitive Symptoms	I find it easy to do things	0.678	0.568	0.465	0.654
	My mind is as clear as it used to be	0.611	0.458	0.373	0.666
Anxiety Symptoms	I have trouble sleeping at night	0.531	0.359	0.313	0.673
Somatic Symptoms	My heart beats faster than usual	0.385	0.278	0.242	0.681
	I get tired for no reason	0.71	0.633	0.313	0.673

^1^ Standardized factor loadings, ^2^ Corrected total-item correlation, ^3^ Cronbach’s Alpha (if Item Deleted).

**Table 7:** Stress level categorization and reliability of the PSS (nine items).

Stress Category	Frequency (%)	Cronbach’s Alpha
Low stress (0–13)	77 (7.7)	0.743
Moderate stress (14–26)	633 (63.2)	
High stress (27–40)	292 (29.1)	

**Table 8:** Association of PSS stress categories with gender, nationality, and education level.

Variable	Category	Low Stress (f, %)	Moderate Stress (f, %)	High Stress (f, %)	*p*-Value
Gender	Male	34 (8)	263 (61.7)	129 (30.3)	N.S
	Female	43 (7.50)	370 (64.2)	163 (28.3)	
Nationality	China	61 (11.7)	374 (71.8)	86 (16.5)	<0.001
	Pakistan	16 (3.3)	259 (53.8)	206 (42.8)	
Current Education Level	Bachelor’s	66 (8.8)	488 (65.2)	194 (25.9)	<0.001
	Master’s	8 (4.6)	108 (62.1)	58 (33.3)	
	Doctoral	3 (3.8)	37 (46.3)	40 (50)	

**Table 9:** Reliability and factor analysis results for Perceived Stress Scale (PSS-9) factors.

Subscale	Items	Cronbach’s Alpha	Inter-Item Correlation	Variance Explained (%)
Perceived Helplessness	P4, P5, P6, P7, P9, P10, P13	0.891	N/A	45.09
Self-Efficacy	P1, P3	N/A	0.609	12.75

**Table 10:** Cultural differences in eating behaviors and psychological health between Chinese and Pakistani students.

Scales (Total Score)	Levene’s Test (F-Statistic, *p*)	t	df	*p*-Value	Mean Difference	95% CI (Lower, Upper)
NIAS	F = 49.506, *p* < 0.001	−9.026	976.34	<0.001	−0.422	(−0.51472, −0.32946)
DOS	F = 132.836, *p* < 0.001	11.136	823.53	<0.001	0.335	(0.27488, 0.39556)
SDS	F = 12.391, *p* < 0.001	10.247	983.64	<0.001	0.229	(0.18556, 0.27326)
PSS	F = 7.282, *p* = 0.007	10.762	984.06	0.007	4.116	(3.35896, 4.87218)

## Data Availability

The data presented in this study are available on request from the corresponding author.

## Supplementary Materials

The following supporting information can be downloaded at: https://www.mdpi.com/article/10.3390/nu17050795/s1, Table S1: Overall ANOVA Results; Table S2: Post-Hoc Results for NIAS and DOS; Table S3: Post-Hoc Results for SDS and PSS; Table S4: Mann-Whitney U Test Results for BMI Categories.

## Abbreviations

The following abbreviations are used in this manuscript:

NIAS	Nine-Item Avoidant/Restrictive Food Intake Disorder Scale
DOS	Düsseldorf Orthorexia Scale
NESQ	Night Eating Syndrome Questionnaire
SDS	Zung’s Self-Rating Depression Scale
PSS	Perceived Stress Scale
CFA	Confirmatory Factor Analysis
SPSS	Statistical Package for the Social Sciences
AMOS	Analysis of Moment Structures
CFI	Comparative Fit Index
TLI	Tucker–Lewis Index
RMSEA	Root Mean Square Error of Approximation
CMIN/DF	Chi-Square Minimum Degrees of Freedom
Cronbach’s α	Cronbach’s Alpha
DF	Degree of freedom
AVE	Average Variance Extracted
CR	Composite Reliability
SE	Standard Error
SFL	Standardized Factor Loading
CI	Confidence Interval
RC	Residual Covariances
SEM	Structural Equation Modeling
