# Supplementary material for: Psychometric Evaluation of Eating Behaviors and Mental Health Among University Students in China and Pakistan: A Cross-Cultural Study
Source: Nutrients. 2025 Feb 25;17(5):795. doi: 10.3390/nu17050795 (PMC11901892; doi:10.3390/nu17050795)
Supplement: Supplementary file 1 [file nutrients-17-00795-s001.zip › nutrients-3493531-supplementary.pdf]

Table S1: Overall ANOVA Results

Measure	F-statistic	df Between	df Within	p-value
NIAS Total Score	11.97	3	998	<0.001
DOS Total Score	0.21	3	998	0.888
SDS Total Score	2.28	3	998	0.078
PSS Total Score	2.63	3	998	0.049

Table S1 provides the results of the ANOVA tests for NIAS, DOS, SDS, and PSS scores. Significant differences were observed for NIAS ($F(3, 998) = 11.97$, $p < 0.001$, $\eta^2 = 0.06$) and PSS ($F(3, 998) = 2.63$, $p = 0.049$, $\eta^2 = 0.01$). These findings suggest that avoidant/restrictive eating behaviors and perceived stress levels vary across BMI groups. However, no significant differences were detected for DOS ($F(3, 998) = 0.21$, $p = 0.888$, $\eta^2 = 0.001$) or SDS ($F(3, 998) = 2.28$, $p = 0.078$, $\eta^2 = 0.007$), indicating consistent orthorexia tendencies and depressive symptoms across BMI categories.

Table S2: Post-Hoc Results for NIAS and DOS

Measure	(I) BMI Group	(J) BMI Group	Mean Difference (I-J)	CI		Sig.
				Lower Bound	Upper Bound	
NIAS Total Score	Underweight	Normal	0.32473	0.15	0.50	0.000
	Underweight	Overweight	0.44793	0.22	0.67	0.000
	Underweight	Obese	0.61353	0.23	0.99	0.000
	Normal	Overweight	-0.12320	-0.30	0.05	0.267
	Normal	Obese	-0.28880	-0.64	0.07	0.157
	Overweight	Obese	-0.16560	-0.55	0.22	0.678
DOS Total Score	Underweight	Normal	-0.01501	-0.13	0.10	0.988
	Underweight	Overweight	-0.04214	-0.19	0.11	0.888
	Underweight	Obese	0.01018	-0.25	0.27	1.000
	Normal	Overweight	-0.02713	-0.15	0.09	0.935
	Normal	Obese	0.02519	-0.21	0.27	0.993
	Overweight	Obese	0.05232	-0.31	0.31	0.953

* Post-hoc comparisons were conducted using Tukey's HSD test.

The ANOVA results (**Table S2**) for the NIAS Total Score reveal significant differences among BMI categories ($F(3, 998) = 11.97$, $p < 0.001$, $\eta^2 = 0.06$), indicating that eating behaviors related to avoidant or restrictive food intake vary across groups. Post-hoc analysis using Tukey's HSD test shows that the Underweight group scored significantly higher compared to the Normal (Mean Difference = 0.32473, 95% CI [0.15, 0.50], $p < 0.001$), Overweight (Mean Difference = 0.44793, 95% CI [0.22, 0.67], $p < 0.001$), and Obese groups (Mean Difference = 0.61353, 95% CI [0.23, 0.99], $p < 0.001$). This suggests that individuals in the Underweight category are more likely to exhibit avoidant or restrictive eating habits.

In contrast, the ANOVA results for the DOS Total Score indicate no significant differences among BMI groups ($F(3, 998) = 0.21$, $p = 0.888$, $\eta^2 = 0.001$). Pairwise comparisons using Tukey's HSD test confirm this, with no significant differences observed between any of the BMI groups. This implies that orthorexia tendencies, which involve extreme focus on healthy eating, are consistent across BMI categories.

Table S3: Post-Hoc Results for SDS and PSS

Measure	(I) BMI Group	(J) BMI Group	Mean Difference (I-J)	CI		Sig.
				Lower Bound	Upper Bound	
SDS Total Score	Underweight	Normal	-0.00187	-0.08	0.08	1.000
	Underweight	Overweight	-0.08120	-0.18	0.03	0.215
	Underweight	Obese	-0.07367	-0.26	0.11	0.736
	Normal	Overweight	-0.07934	-0.16	0.00	0.077
	Normal	Obese	-0.07180	-0.24	0.10	0.708
	Overweight	Obese	0.00753	-0.18	0.19	1.000
PSS Total Score	Underweight	Normal	-0.68721	-2.16	0.79	0.627
	Underweight	Overweight	-1.95182	-3.82	-0.08	0.037
	Underweight	Obese	-1.35887	-4.56	1.84	0.695
	Normal	Overweight	-1.26461	-2.73	0.21	0.120
	Normal	Obese	-0.67166	-3.66	2.32	0.939
	Overweight	Obese	0.59295	-2.61	3.80	0.964

* Post-hoc comparisons for SDS and PSS were conducted using Tukey's HSD test.

Interpretation of Table 4: Post-Hoc Results for SDS and PSS

The ANOVA results for the PSS Total Score revealed significant differences among BMI groups ($F(3, 998) = 2.63$, $p = 0.049$, $\eta^2 = 0.01$). Post-hoc analysis using Tukey's HSD test showed that the Underweight group had significantly lower PSS scores compared to the Overweight group (Mean Difference = -1.95182, 95% CI [-3.82, -0.08], $p = 0.037$), suggesting that underweight individuals perceive less stress than overweight individuals.
No significant differences were found in other pairwise comparisons, indicating largely consistent stress levels across the remaining BMI groups. For the SDS Total Score, the ANOVA results showed a trend towards significance ($F(3, 998) = 2.28$, $p = 0.078$, $\eta^2 = 0.007$), but post-hoc analysis revealed no significant differences between specific BMI groups. This suggests that depressive symptoms are relatively stable across BMI categories.

Table S4: Mann-Whitney U Test Results for BMI Categories

Comparison	Scale	U Statistic	Z Score	p-value
**Underweight vs Normal**	NIAS	40,941.50	-3.852	<0.001
	DOS	50,597.00	-0.181	0.856
	SDS	50,676.50	-0.15	0.88
	PSS	48,292.00	-1.057	0.29
**Underweight vs Overweight**	NIAS	8,296.00	-4.793	<0.001
	DOS	11,449.00	-0.811	0.418
	SDS	10,545.50	-1.951	0.051
	PSS	10,047.00	-2.581	0.01
**Underweight vs Obese**	NIAS	1,448.50	-3.707	<0.001
	DOS	2,366.00	-0.41	0.682
	SDS	2,246.50	-0.839	0.402

	PSS	2,089.00	-1.405	0.16
**Normal vs Overweight**	NIAS	44,974.50	-2.435	0.015
	DOS	49,135.50	-0.859	0.39
	SDS	45,468.00	-2.247	0.025
	PSS	45,423.00	-2.265	0.024
**Normal vs Obese**	NIAS	8,240.00	-2.093	0.036
	DOS	10,103.00	-0.401	0.689
	SDS	9,534.50	-0.916	0.36
	PSS	9,371.00	-1.065	0.287
**Overweight vs Obese**	NIAS	2,320.00	-0.629	0.529
	DOS	2,485.50	-0.038	0.97
	SDS	2,471.50	-0.087	0.93
	PSS	2,456.00	-0.143	0.886

Note: Significant p-values ($p < 0.05$) are typically bolded to highlight statistically significant differences.

- **NIAS**: Statistically significant differences were observed across most BMI category comparisons. Participants in the Underweight group exhibited higher NIAS scores compared to other groups (e.g., Underweight vs Normal: $U = 40,941.5$, $p < 0.001$).
- **PSS**: Significant differences in stress levels were found between Underweight vs Overweight ($U = 10,047.0$, $p = 0.010$) and Normal vs Overweight ($U = 45,423.0$, $p = 0.024$).
